# A Rare Case of Simultaneous Histoplasmosis and Coccidioidomycosis

**DOI:** 10.7759/cureus.67145

**Published:** 2024-08-18

**Authors:** Nikeith Shah, Michelle Manikkam, Hamid Parakhoodi

**Affiliations:** 1 Internal Medicine, WellSpan York Hospital, York, USA; 2 Infectious Diseases, WellSpan York Hospital, York, USA

**Keywords:** amphotericin, coinfection, infectious disease, fungal infection, coccidioides, histoplasma, coccidioidomycosis, histoplasmosis

## Abstract

*Histoplasma *and *Coccidioides *are fungi that can cause serious infections in immunocompromised patients. *Histoplasma *is primarily endemic to the central and eastern United States, while *Coccidioides *is primarily endemic to the southwestern United States. Here, we present a case of simultaneous histoplasmosis and coccidioidomycosis. A 69-year-old female with a past medical history of rheumatoid arthritis and polymyalgia rheumatica on immunosuppression presented to the emergency department (ED) with fevers, malaise, and confusion. She initially developed these symptoms a month prior while visiting her son in Tennessee. During this time, she lived in his basement where mold exposure was confirmed. Her symptoms gradually improved but recurred, prompting her to come to the ED. In the ED, her vital signs were as follows: temperature of 36.5˚C, heart rate of 88, respiratory rate of 16, blood pressure of 158/88, and oxygen saturation of 94% on room air. She was alert and oriented without focal neurologic deficits. Heart sounds were regular rate and rhythm, lungs were clear to auscultation bilaterally and abdomen was soft, non-tender, and non-distended. No skin rashes were observed either. Laboratory work revealed an elevated C-reactive protein (CRP), thrombocytopenia, and transaminitis. Chest X-ray showed patchy airspace disease in the left lower lobe, and she underwent a lumbar puncture which was negative for meningitis. Due to her travel to Tennessee, a urine *Histoplasma* antigen test was ordered which resulted positive, along with a beta-1,3-D-glucan level >500 picograms per milliliter (pg/mL), indicating disseminated histoplasmosis. *Coccidioides* antibodies also resulted positive, pointing to concurrent coccidioidomycosis. The patient was subsequently started on intravenous amphotericin B. Over the following days, the patient’s transaminitis and thrombocytopenia improved, and she was ultimately discharged on oral itraconazole with outpatient infectious disease follow-up. Although the patient’s exposure to mold was likely the source of her histoplasmosis, the source of her coccidioidomycosis is less clear given its endemicity. Even rarer is the coinciding infections, and to the best of our knowledge, this is one of the very few known cases. Immunocompromised patients who present with infectious symptoms should have a low threshold for a fungal infection workup, as prompt treatment is crucial to limiting the morbidity and mortality of these infections. Furthermore, geographic location should not narrow one’s workup to endemic fungi only, as evidenced by this patient's simultaneous infections.

## Introduction

Histoplasmosis is an infection caused by the fungus *Histoplasma capsulatum* (*H. capsulatum*), and coccidioidomycosis is an infection caused by the fungus *Coccidioides immitis* (*C. immitis*) or *Coccidioides posadasii* (*C. posadasii*). In the United States (U.S.), *H. capsulatum* is endemic to the central and eastern regions [1], while *C. immitis* and *C. posadasii* are endemic to the southwestern region [2]. Both infections are contracted primarily via inhalation of spores from disturbed soil. Once the spores are inhaled, the fungi transform from a mold to a yeast, leading to subsequent localized or systemic infections. Common clinical symptoms of both diseases include fevers, night sweats, cough, myalgias, and arthralgias. Immunocompromised patients, specifically those who are neutropenic, are at an increased risk for fungal infections such as histoplasmosis and coccidioidomycosis [3]. Neither disease is contagious, but they have rarely been shown to spread via organ transplant [1,4,5]. The last reported data by the Centers for Disease Control and Prevention (CDC) from 2021 showed 1,459 cases of histoplasmosis and 20,320 cases of coccidioidomycosis in the U.S., but there is not much data on coinciding infections [6]. Here, we present a case of simultaneous histoplasmosis and coccidioidomycosis.

## Case presentation

**Figure 1:**
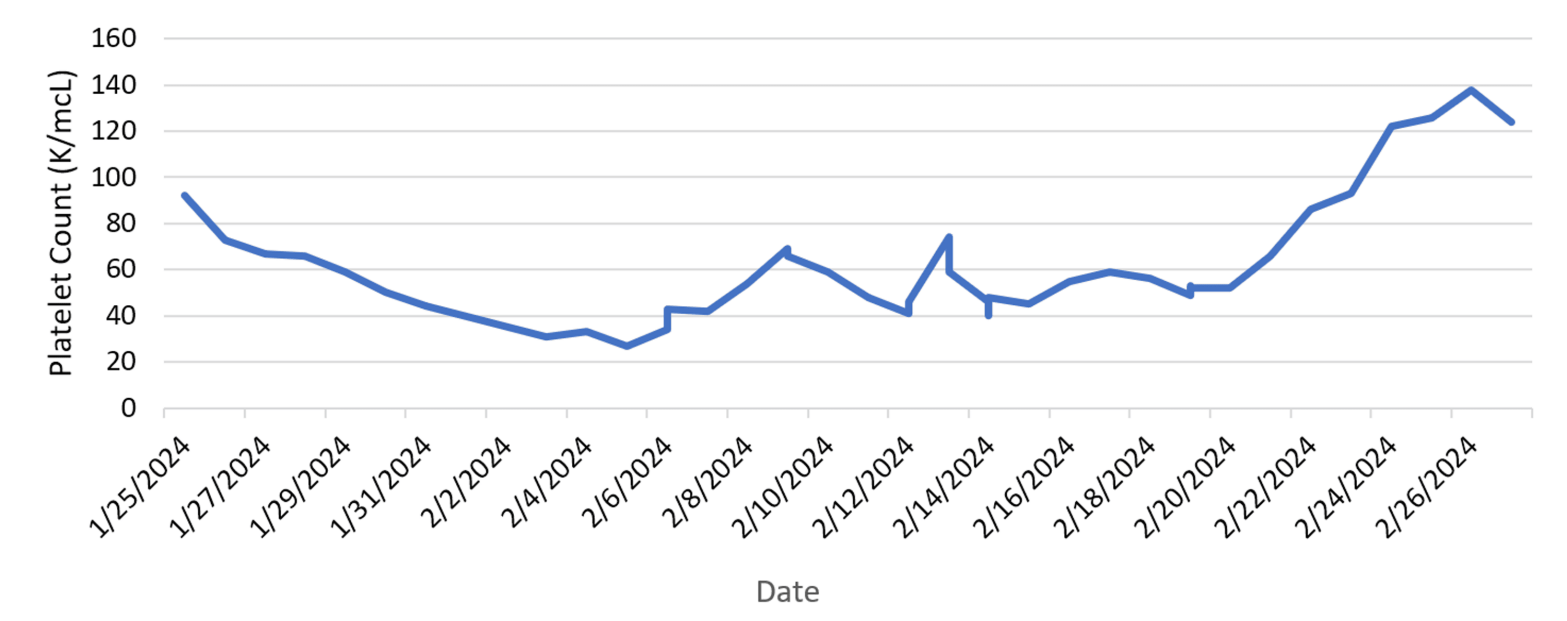
Platelet count during the hospital course.

A 69-year-old female with a past medical history of rheumatoid arthritis and polymyalgia rheumatica on infliximab and methotrexate, hypothyroidism, and type 2 diabetes presented to the emergency department (ED) with a chief complaint of fever, headaches, cough, and night sweats. Her symptoms began approximately 10 days before presentation, prompting a visit to the ED three days prior. She was ultimately diagnosed with pneumonia and was treated with Levaquin. Her symptoms failed to improve, however, and she began developing intermittent confusion and myalgias, requiring a return to the hospital. Of note, a month before presentation, she had traveled to Tennessee to visit her son. During this time, she and her son developed fever, cough, congestion, and chills which resolved after a week. She was living in her son’s basement while there, and there was known mold exposure. She had denied exposure to birds or bats, recent cave exploration, or disturbing the local soil. She primarily lived in Pennsylvania, with intermittent trips to Tennessee. On admission, the patient was afebrile and all vital signs were stable. Physical exam was benign, with no significant cardiopulmonary or neurological exam findings, and no signs of meningeal irritation. A complete blood count revealed a white blood cell count of 11.8 thousand cells per microliter (K/mcL) of blood (normal range: 4.0 - 11.0 K/mcL) and a platelet count of 92 K/mcL (normal range: 140 - 400 K/mcL) (Figure 1).

**Figure 2:**
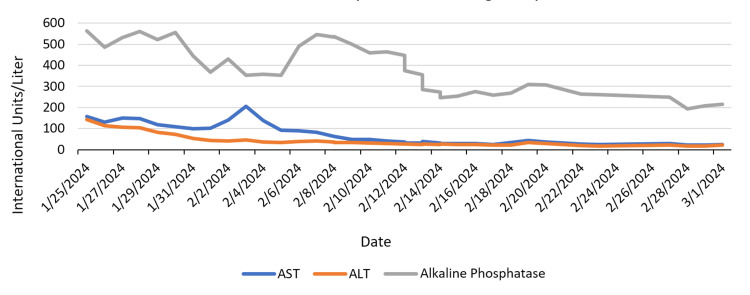
AST, ALT, and alkaline phosphatase levels during the hospital course. AST, aspartate transaminase; AST, alanine transaminase.

A comprehensive metabolic panel revealed an aspartate transaminase (AST) of 158 international units per liter (IU/L) (normal range: 13 - 39 IU/L) and alanine transaminase (ALT) of 142 IU/L (normal range: 7 - 52 IU/L) (Figure 2).

**Figure 3:**
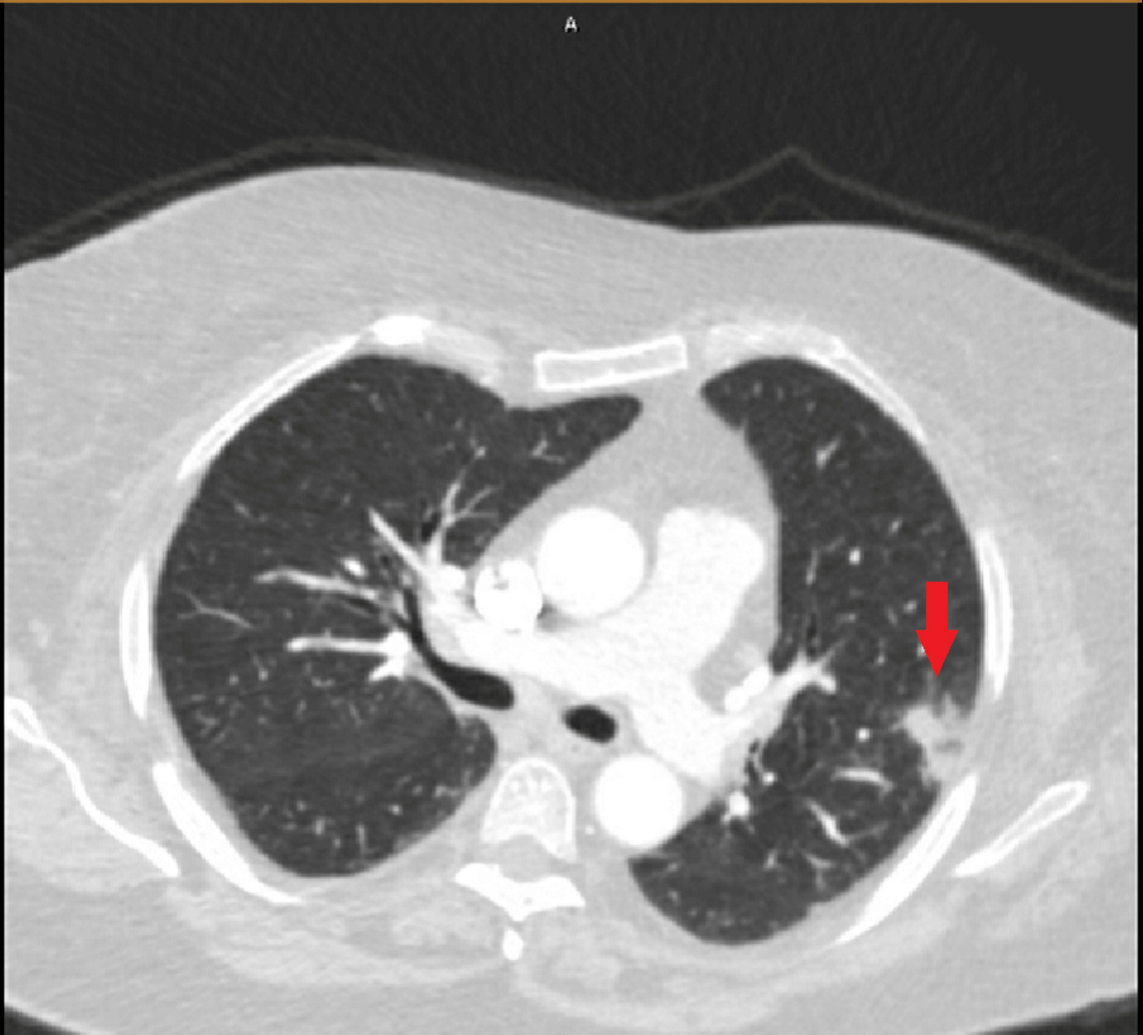
CT chest with contrast showing focal airspace consolidation in the periphery of the left upper lobe (shown by red arrow) suggestive of focal pneumonia.

Other laboratory work was significant for a C-reactive protein (CRP) of 42.2 milligrams per liter (mg/L) (normal range: 0.0 - 10.0 mg/L), procalcitonin of 0.25 nanograms per milliliter (ng/mL) (normal range: 0.0 - 0.25 ng/mL) and lactate of 1.2 millimoles per liter (mmol/L) (normal range: 0.5 - 2.0 mmol/L). Computed tomography (CT) scans of the head, abdomen, and pelvis were unremarkable, and a CT of the chest performed three days prior showed a left upper lobe focal airspace consolidation (Figure 3).

**Figure 4:**
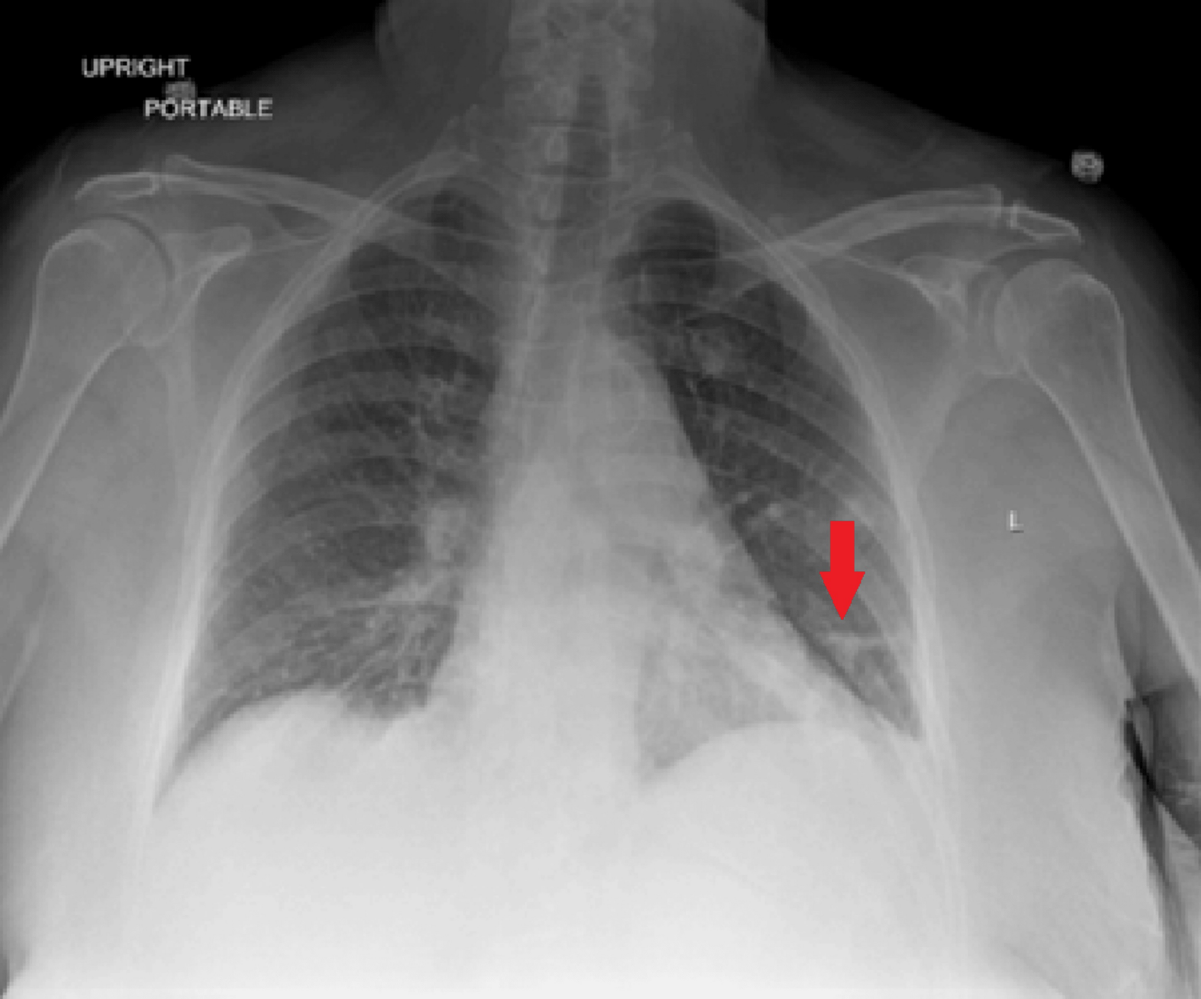
Chest X-ray showing worsening of the left lower lobe infiltrate (shown by red arrow).

A chest X-ray on admission this visit showed worsening of the left lung infiltrate (Figure 4).

The infectious diseases (ID) team was consulted, and given the patient’s symptoms and laboratory work in the setting of known immunosuppression, there was high concern for an opportunistic fungal infection. A variety of infectious blood tests were obtained, including a tickborne panel, herpes simplex virus (HSV) polymerase chain reaction (PCR), cytomegalovirus (CMV) PCR, and *Toxoplasma* antibodies, all of which resulted negative. A lumbar puncture was also performed, and the meningitis-encephalitis panel returned negative. Fungal workup was initiated, including beta-1,3-D-glucan, urine *Histoplasma* antigen, and *Coccidioides* antibodies. Due to the patient’s transaminitis, the gastroenterology team was consulted, and the patient underwent an endoscopic ultrasound which did not show choledocholithiasis or intra and extrahepatic dilation. The beta-1,3-D-glucan level was greater than 500 picograms per milliliter (pg/mL), and the urine *Histoplasma* antigen test was positive, indicating disseminated histoplasmosis. The *Coccidioides* antibody also resulted positive, pointing to concurrent coccidioidomycosis. The patient was subsequently initiated on intravenous amphotericin B. Bronchoalveolar lavage was performed twice; however, the specimens were unsatisfactory for evaluation due to the sub-optimal quantity of pulmonary macrophages. The patient’s transaminitis and thrombocytopenia slowly improved, and the patient was ultimately discharged on a three-month course of oral itraconazole.

## Discussion

The patient’s development of both fungal infections can be analyzed through a variety of angles. Given the patient was on two different immunosuppressants, she was inherently at an increased risk for contracting fungal infections. Studies have shown that immunosuppressants reduce neutrophil function, which is important in fighting fungal infections [7]. Interestingly, this patient was not neutropenic; nonetheless, this does not exclude her risk of becoming infected. There have been multiple reports in the literature describing fungal infections in non-neutropenic patients, though it should be noted that these incidents primarily involve critically ill patients [8,9]. The patient’s recent travel to Tennessee also presented a clear risk factor for contracting *Histoplasma*, as the fungus is known to be endemic to this area. She did not have any of the other typical risk factors for contracting *Histoplasma *during her time there, such as exposure to birds or bats, cave exploration, or disturbing the local soil. She was, however, living in her son’s basement where there was a known mold exposure; this was likely the source of her infection. In regard to the patient’s coinciding infection with *Coccidioides*, she denied recent travel to the southwestern region of the United States. However, a 2023 retrospective analysis of over 123,000 patients diagnosed with either histoplasmosis, blastomycosis, or coccidioidomycosis found that many of these cases were not located in their historically portrayed regions of endemicity [10]. Moreover, there have been reported cases of histoplasmosis in California, further pointing to the fact that these species of fungi are not geographically restricted to where they once were believed to be [11,12]. Histoplasmosis and coccidioidomycosis have also been seen in animals, particularly dogs and cats [13]. The patient did endorse owning both a dog and a cat; however, studies have not shown that these infections can be transmitted from animals to humans [1]. Although we do not believe this patient contracted the infection from her pets, perhaps more research is needed to confirm that these infections cannot be contracted from animals. The most striking aspect of this case is the concurrent infection with both fungi. To the best of our knowledge, there is only one previously documented case of histoplasmosis and coccidioidomycosis, which was in 1965 [14]. The uniqueness of such an incidence makes this an even more interesting case. It is possible that simultaneous infections are occurring more frequently, but clinicians may only be performing fungal workups based on the specific region of a patient.

## Conclusions

This case is of great significance as it adds to the sparse literature on simultaneous *Histoplasma *and *Coccidioides *infections. Further research needs to be conducted on the rarity of this occurrence. It is also clear that extensive history taking, including the patient’s immune status, travel history, housing circumstances, and daily activities, is of utmost importance when determining the risk of contracting a fungal infection. Immunocompromised patients who present with signs or symptoms of an infection should have a low threshold for a fungal infection workup. Although *Histoplasma *and *Coccidioides *are not primarily endemic to the same regions, clinicians should have a low threshold for working up both infections regardless of the patient’s recent travel history or current geographic location.

## References

[1] Centers for Disease Control and Prevention (2024). Histoplasmosis. Centers for Disease Control and Prevention.

[2] Galgiani JN, Ampel NM, Blair JE (2016). Executive summary: 2016 Infectious Diseases Society of America (IDSA) clinical practice guideline for the treatment of coccidioidomycosis. Clin Infect Dis.

[3] Herbrecht R, Neuville S, Letscher-Bru V, Natarajan-Amé S, Lortholary O (2000). Fungal infections in patients with neutropenia: challenges in prophylaxis and treatment. Drugs Aging.

[4] Centers for Disease Control and Prevention (2024). Valley fever (coccidioidomycosis). Centers for Disease Control and Prevention.

[5] Roy M, Park BJ, Chiller TM (2010). Donor-derived fungal infections in transplant patients. Curr Fungal Infec Rep.

[6] (2024). Surveillance for coccidioidomycosis, histoplasmosis, and blastomycosis during the COVID-19 pandemic - United States, 2019-2021. https://www.cdc.gov/mmwr/volumes/73/wr/mm7311a2.htm.

[7] Vymazal O, Bendíčková K, De Zuani M, Vlková M, Hortová-Kohoutková M, Frič J (2021). Immunosuppression affects neutrophil functions: does calcineurin-NFAT signaling matter?. Front Immunol.

[8] Cortegiani A, Russotto V, Maggiore A, Attanasio M, Naro AR, Raineri SM, Giarratano A (2016). Antifungal agents for preventing fungal infections in non-neutropenic critically ill patients. Cochrane Database Syst Rev.

[9] Azim A, Ahmed A (2024). Diagnosis and management of invasive fungal diseases in non-neutropenic ICU patients, with focus on candidiasis and aspergillosis: a comprehensive review. Front Cell Infect Microbiol.

[10] Mazi PB, Sahrmann JM, Olsen MA (2023). The geographic distribution of dimorphic mycoses in the United States for the modern era. Clin Infect Dis.

[11] Ghadiya K, Dunn R, Singh G, Lai H, Garcia-Pacheco R (2023). Disseminated histoplasmosis in central California seen in an immunocompromised patient. J Investig Med High Impact Case Rep.

[12] Govindarajan A, Sous R, Venter F (2023). A case of disseminated histoplasmosis from California, in the setting of secondary hemophagocytic lymphohistiocytosis: a diagnostic challenge. J Investig Med High Impact Case Rep.

[13] Brömel C, Sykes JE (2005). Histoplasmosis in dogs and cats. Clin Tech Small Anim Pract.

[14] Perry LV, Jenkins DE, Whitcomb FC (1965). Simultaneously occurring pulmonary coccidioidomycosis and histoplasmosis. Am Rev Respir Dis.

